# Impact of respiratory viral infections during pregnancy on the neurological outcomes of the newborn: current knowledge

**DOI:** 10.3389/fnins.2023.1320319

**Published:** 2024-01-08

**Authors:** Sara Manti, Giulia Spoto, Antonio Gennaro Nicotera, Gabriella Di Rosa, Giovanni Piedimonte

**Affiliations:** ^1^Pediatric Unit, Department of Human Pathology in Adult and Developmental Age “Gaetano Barresi”, University of Messina, Messina, Italy; ^2^Unit of Child Neurology and Psychiatry, Department of Biomedical and Dental Sciences and of Morphological and Functional Imaging, University of Messina, Messina, Italy; ^3^Unit of Child Neurology and Psychiatry, Department of Human Pathology in Adult and Developmental Age “Gaetano Barresi”, University of Messina, Messina, Italy; ^4^Department of Pediatrics, Biochemistry and Molecular Biology, Tulane University, New Orleans, LA, United States

**Keywords:** influenza virus, maternal infection, neurodevelopmental outcome, respiratory syncytial virus, respiratory viral infections, severe acute respiratory syndrome coronavirus 2

## Abstract

Brain development is a complex process that begins during pregnancy, and the events occurring during this sensitive period can affect the offspring’s neurodevelopmental outcomes. Respiratory viral infections are frequently reported in pregnant women, and, in the last few decades, they have been related to numerous neuropsychiatric sequelae. Respiratory viruses can disrupt brain development by directly invading the fetal circulation through vertical transmission or inducing neuroinflammation through the maternal immune activation and production of inflammatory cytokines. Influenza virus gestational infection has been consistently associated with psychotic disorders, such as schizophrenia and autism spectrum disorder, while the recent pandemic raised some concerns regarding the effects of severe acute respiratory syndrome coronavirus 2 on neurodevelopmental outcomes of children born to affected mothers. In addition, emerging evidence supports the possible role of respiratory syncytial virus infection as a risk factor for adverse neuropsychiatric consequences. Understanding the mechanisms underlying developmental dysfunction allows for improving preventive strategies, early diagnosis, and prompt interventions.

## 1 Introduction

The human brain, constituted of 85 billion neurons and 85 billion non-neuronal cells, is a complex structure responsible for higher cognitive and behavioral abilities (i.e., perception, memories, thoughts, and language; Bystron et al., 2008; Azevedo et al., 2009).

The central nervous system (CNS) development mainly occurs during the prenatal period, and most cerebral neurogenesis happens within the 28th gestational week (Davis et al., 2011; Hadders-Algra, 2021); therefore, pregnancy is considered a vulnerable epoch for the neuropsychiatric outcome of the offspring since genetic, epigenetic, and environmental factors may disrupt the typical developmental trajectory (Hadders-Algra, 2021; San Martín-González et al., 2023).

The intrauterine environment represents the first influence on the fetus’s development, and maternal health has long been considered an important factor impacting the child’s well-being (Barker, 1990).

According to the Developmental Origins of Health and Disease hypothesis, events occurring during this sensitive period might have short- and long-term consequences on the fetus epigenetic program, depending on the timing and duration of exposure to the *noxa* (Davis et al., 2011; Palma-Gudiel et al., 2019).

Numerous studies proved that neurotropic viral agents may cross the placental barrier and that *in utero* exposure may severely affect fetal brain development (Zimmer et al., 2021). Depending on the specific virus, the timing of infection during gestation, and the efficiency of viral neutralization and clearance by the host immune system, neuropsychiatric sequelae can range from mild to severe, and even compromise fetal viability (Gordon-Lipkin et al., 2021; Manti et al., 2022). Additionally, the maternal immune response, including the synthesis of pro-inflammatory cytokines, as well as the fetal pro-inflammatory mediators, can sustain the neuroinflammation affecting the CNS development in the fetus (Han et al., 2021; Elgueta et al., 2022; Kwon et al., 2022).

Consistent literature data reported the association between prenatal risk factors, such as *in utero* infections, and diseases in childhood and adulthood, including neuropsychiatric disorders (Zimmer et al., 2021; San Martín-González et al., 2023). In line with this theory, Torrey and Peterson proposed the “Viral Hypothesis of Schizophrenia,” according to which a viral infection acquired during prenatal life could be involved in schizophrenia development (Torrey and Peterson, 1976). Subsequently, several reports confirmed an increased risk for schizophrenia onset in patients born by women affected by viral infection during pregnancy, especially in subjects born during late winter and spring, seasons during which Influenza infection is primarily detected (Hare et al., 1972; Machón et al., 1987; Mednick et al., 1988; O’Callaghan et al., 1991; Adams et al., 1993; Mednick et al., 1994; Kunugi et al., 1995; Izumoto et al., 1999; Limosin et al., 2003; Brown, 2006; Fatemi et al., 2012). In addition, further research highlighted the occurrence of neurodevelopmental disorders, such as Autism Spectrum Disorder (ASD), Attention-Deficit/Hyperactivity Disorder (ADHD), microcephaly, epilepsy, cognitive impairment, and bipolar disorder in children with a history of prenatal maternal infections (Jiang et al., 2016; Tioleco et al., 2021; Elgueta et al., 2022; Walle et al., 2022; Zhu et al., 2022).

In the past, respiratory viruses were not considered highly neurotropic agents, and most authors did not heed the possible vertical transmission to the fetus (Zimmer et al., 2021; San Martín-González et al., 2023). However, respiratory viral infections are the most frequently reported during pregnancy, and the recent pandemics raised some concerns regarding the neurodevelopmental consequences for the offspring of women affected by respiratory infections (San Martín-González et al., 2023; Sturrock et al., 2023).

**Figure 1:**
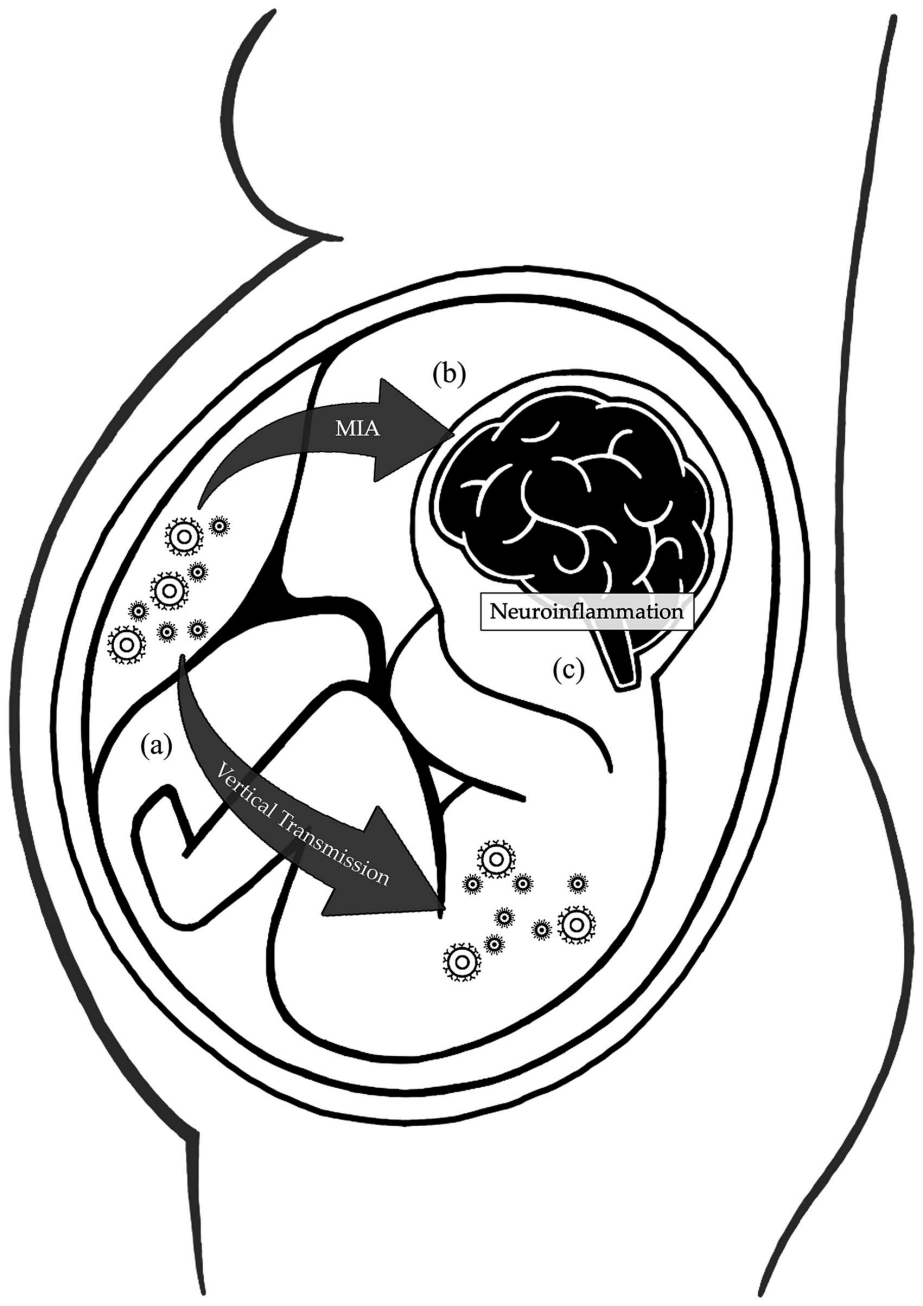
Mechanisms affecting the brain development during maternal respiratory viral infections. During pregnancy, physiological changes in the maternal immune system increase the susceptibility to respiratory viral infections. **(A)** The reduction in the Th1/Th2 phenotype ratio and natural killer cell activation impair the immune response, while the intravascular inflammation enhances the virulence, increasing the risk of vertical transmission. **(B)** Cytokines and inflammatory mediators produced by the mother during infections can cross the placental barrier and interfere with the fetal brain development. **(C)** Both the vertical transmission and maternal immune activation induce neuroinflammation, further sustained by fetal production of inflammatory products. All these mechanisms disrupt the development of the fetal central nervous system.

This review aims to investigate the impact of prenatal exposure to maternal respiratory infections on neuropsychiatric outcomes throughout the epochs of life. In order to achieve this goal, we summarized what is known about the early stages of brain development and the systemic changes, including immune activation, occurring in pregnant women affected by respiratory viral infections, such as Influenza virus, severe acute respiratory syndrome coronavirus 2 (SARS-CoV-2), Respiratory Syncytial virus (RSV), and other respiratory viruses (Figure 1). Finally, we reviewed literature data focusing on neuropsychiatric disorders associated with common respiratory viral infections, including Influenza virus, SARS-CoV-2, RSV, and other respiratory viruses.

## 2 Central nervous system development during pregnancy

Brain development is a process that starts during the early weeks of the pregnancy and requires about 40 years to reach a mature configuration (Hadders-Algra, 2021). However, gestation is an epoch of profound changes in the CNS, and it is widely accepted that prenatal experiences can influence the developing motor and sensory-perceptual systems, affecting the neurobehavioral and cognitive outcomes of the offspring (Kostović et al., 1995; Han et al., 2021). During pregnancy, brain development consists of four main processes, including neuronal proliferation and migration, mainly occurring during the embryonic and early fetal periods, synaptogenesis and myelination, which start during the intrauterine life and continue through the postnatal age (Kostović et al., 1995; Borsani et al., 2019).

By the 2–3th gestational week, the ectoderm initiates a process of folding and fusion that culminates in the neural tube formation at embryonic day 30. The rostral portion of the neural tube gives rise to the prosencephalic, mesencephalic and romboencephalic vesicles, namely the precursors of the forebrain, midbrain, and hindbrain. The prosencephalon will originate the diencephalon (subcortical structures) and the telencephalon (cerebral cortices; Bystron et al., 2008; Borsani et al., 2019). Neurogenesis begins after the complete closure of the neural tube, approximately at embryonic day 33, in the ventricular zone (VZ). Neuronal and glial progenitors, named neural stem/precursor cells (NPCs), arise from the VZ and their proliferation results in a new layer, called subventricular zone or germinal matrix (Bystron et al., 2008; Elgueta et al., 2022). Most cortical neurons emerge from this last region and form the preplate, that together with the marginal zone (near the pial surface) and the subplate zone (near the ventricle), constitutes the primordial cerebral cortex (Kostović et al., 1995; Borsani et al., 2019). Notably, the subplate is a transient layer rich in neuronal fibers and represents the primary site of neuronal differentiation and synaptogenesis (Hadders-Algra, 2021).

The cerebellum, which contains 80% of the brain neurons, also originates between the end of the first and the beginning of the second trimester of pregnancy. At 11 weeks of gestational age, the VZ will give rise to neurons constituting the future deep cerebellar nuclei and the Purkinje cells. Moreover, the cerebellar anlage has its transient area, known as external granular layer. Since the 15^th^ week of gestational age, it will generate the granule cells and disappear after the first post-term year (Amore et al., 2021).

Neuronal migration and myelination begin around the 20th week of gestational age and occur with a spatio-temporal gradient: the former takes place slightly earlier in the occipital areas than in the frontal ones, the latter starts in the subcortical regions before the cortical ones (Trivedi et al., 2009; Tau and Peterson, 2010). Moreover, neuronal migration usually ends within 26–29 weeks of gestational age; on the contrary, myelination of the corticospinal tracts lasts until 2–3 years of postnatal life (Borsani et al., 2019).

During the third trimester of pregnancy, neuronal proliferation gradually declines while glial cell production continues, inducing an increase in the volume of the white matter. In this period, the brain reaches a full-blown adult configuration, presenting an immature cytoarchitecture. In particular, neurotransmitter systems have developed, and neuronal differentiation, axonal ingrowth, and synaptogenesis processes become more intense (Hadders-Algra, 2021).

In addition, depending on the cortical region, area differentiation begins between the 24th and the 34th gestational weeks, persisting until the term age (Kostović et al., 1995; Borsani et al., 2019).

## 3 Pregnancy-related immune effects on the maternal organism

Pregnancy induces significant physiological changes in the mother, involving the immune, respiratory, cardiovascular, and hormonal systems (Manti et al., 2022). Since there is no vascular continuity between the mother and the fetus, the placenta plays a fundamental role in the maternal-fetal exchanges (Veenstra van Nieuwenhoven et al., 2003). It allows the transport of nutrients and antibodies from the maternal circulation through membrane transporters and endocytosis, production of hormones, and waste removal from the fetal blood. In addition, it represents a barrier from pathogens, toxicants, and teratogens (Gordon-Lipkin et al., 2021).

Recent proof of trans-syncytial nanopores in the human placenta demonstrated that potentially harmful molecules can cross the placenta through paracellular diffusion and adversely affect fetal development (Lewis et al., 2022).

The fetus and placenta have been historically considered an allograft since they express “non-self” paternal antigens. Moreover, the placenta expresses tissue-specific antigens that might potentially trigger maternal immune activation, and data from the literature confirm systemic immune responses to numerous pathogens in animal models and pregnant women (Erlebacher, 2013). At the same time, mechanisms involved in fetomaternal tolerance also prevent pregnancy-associated complications, such as prematurity, preeclampsia, fetal growth restriction, and miscarriages (Kwon et al., 2022). All this evidence proves that pregnancy must not be considered a classical transplantation; rather, it represents a unique immunological condition in which immune activation is locally regulated and limited to the maternal-fetal interface (Saito, 2022).

Nevertheless, the maternal immune system incurs numerous adjustments during gestation that may increase the risk of infections. CD4+ T cell population shows a reduction in the Th1/Th2 phenotype ratio, which allows the pregnancy to progress but can affect the clearance of infected cells (Parkin and Cohen, 2001; Veenstra van Nieuwenhoven et al., 2003). Some authors suggested that this disproportion could be caused by a suppression of maternal T cells by progesterone and fetal regulatory T cells (Saito, 2022). Dendritic cells (DCs), the most potent antigen-presenting cells, have also been implicated in regulating of the Th1/Th2 phenotype ratio (Veenstra van Nieuwenhoven et al., 2003). Moreover, it has been proposed that paternal/fetal antigens are not directly presented by fetal antigen-presenting cells but by maternal DCs, to reduce the probability of acute transplant rejection. Furthermore, maternal DCs are suggested to be trapped in the uterus, minimizing fetal antigen presentation to the lymph nodes (Saito, 2022). This hypothesis is supported by studies on mice models that proved the inability of DCs to migrate from the decidua to lymphatic vessels of the uterus, even after immune stimulation (Erlebacher, 2013).

The fetal regulatory T cells may also reduce the activation of natural killer (NK) cells, which are embryotoxic in pregnant women (Veenstra van Nieuwenhoven et al., 2003; Saito, 2022). Both DCs and NK cells are fundamental mediators of the immune response to viral infections and their reduction can expose the mother to an increased risk of infection (Parkin and Cohen, 2001; Veenstra van Nieuwenhoven et al., 2003).

Besides the immunological effects, pregnancy may induce other systemic modifications that increase susceptibility to viral respiratory infections. Pulmonary compliance is reduced due to diaphragm elevation and shape alterations of the chest, affecting the airway clearance of pathogens. Furthermore, increased intravascular inflammation, with coagulation and fibrinolytic factors circulation, can enhance virulence (Manti et al., 2022).

All these collective mechanisms concur in the onset and evolution of maternal infections, particularly respiratory viral ones, and their spreading to the fetal circulation (Veenstra van Nieuwenhoven et al., 2003; Gordon-Lipkin et al., 2021; San Martín-González et al., 2023). Vertical transmission from the mother to the fetus has been described for neurotropic and non-neurotropic viruses, which can cause the invasion of neural cells and microglia in the developing brain (Zimmer et al., 2021; Manti et al., 2022). Moreover, viral agents can affect the newly formed CNS by interfering with NPCs generation, progenitors of neurons, astrocytes, and oligodendrocytes (Elgueta et al., 2022).

## 4 Maternal immune activation and brain development

**Figure 2:**
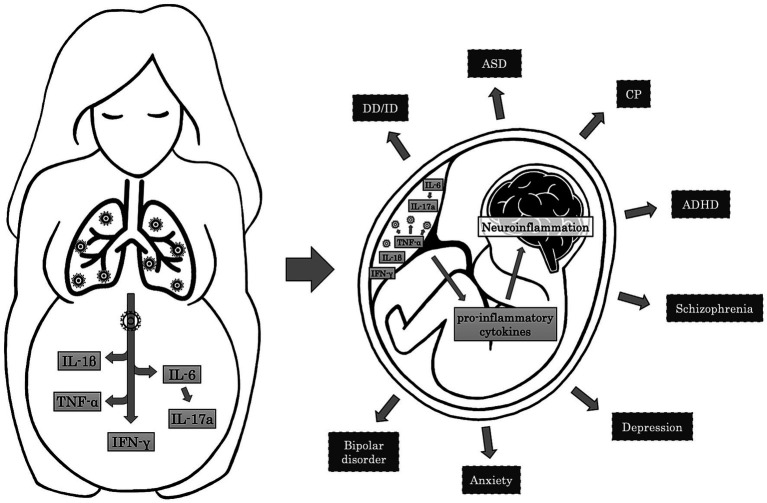
Mechanisms and effects of Maternal Immune Activation on fetal brain. The maternal immune response to respiratory infections induces the release of pro-inflammatory cytokines such as IL-6, IL-1β, TNF-α, and IFN-γ. In turn, IL-6 leads to the activation of Th17 cells and the production of IL-17a. In the placenta, TNF-α contributes to the activation of the immune resident cells, resulting in further production of pro-inflammatory cytokines that cross the placental-fetal barrier and participate in the neuroinflammatory processes in the fetal brain. Neuroinflammation may alter normal brain development, resulting in adverse neuropsychiatric outcomes during childhood and adulthood, such as developmental delay/intellectual disability (DD/ID), autism spectrum disorder (ASD), cerebral palsy (CP), attention-deficit/hyperactivity disorder (ADHD), schizophrenia, depression, anxiety, and bipolar disorder.

Alternatively to vertical transmission, accumulating evidence in the literature supports the hypothesis that maternal immune activation (MIA) constitutes one of the crucial mechanisms through which prenatal infections exert their effects on neurodevelopment (Han et al., 2021; Kwon et al., 2022; Woods et al., 2023; Figure 2). Maternal systemic immune response against infections leads to the production of cytokines and inflammatory mediators, such as interleukin (IL)-6, IL-17a, IL-1β, tumor necrosis factor (TNF)-α, and interferon (IFN)-γ, which may cross the placental barrier depending on the gestational age by diffusion through paracellular trans-trophoblastic water-filled channels (Kwon et al., 2022; Woods et al., 2023). In a rat model, Dahlgren and colleagues proved that the transfer of IL-6 to the fetal circulation was significantly higher during mid-gestation than in late pregnancy (Dahlgren et al., 2006). On the contrary, in a different rat model of MIA, traced IL-1β showed minimal fetal passage during the late gestational period (Girard and Sebire, 2016). These inflammatory factors can affect NPCs development, resulting in cortical defects and neurodevelopmental disorders. Particularly, it has been proved that IL-1β influences neuronal migration, and IL-6 affects the temporal regulation of neurogenesis and reduces the neuronal pool within the cortical layers, promoting an early switch to gliogenic cells and inducing an alteration in the ratio of the neuronal/glial cells (Sloan and Barres, 2014; Woods et al., 2023). Additionally, IL-6 induces differentiation of naïve T cells to a Th17 phenotype, producing IL-17a, a cytokine that disorganises the cortical cytoarchitecture. These cortical alterations are associated with ASD-like behaviors in the offspring, including abnormal ultrasonic vocalization, a deficit in social interaction, and repetitive behavior (Elgueta et al., 2022). Proinflammatory cytokines can also affect myelination by inhibiting the proliferation and survival of oligodendrocyte progenitors, a cellular type that usually persists later in life and can differentiate even in adulthood (Taylor et al., 2010; Favrais et al., 2011).

The pool of NPCs is further controlled by the microglia, which are also responsible for antigen presentation, neuronal remodelling, and synaptic plasticity (Elgueta et al., 2022). In addition, fetal microglia activation may sustain neuroinflammation and impair neurodevelopment by secreting inflammatory products (i.e., TNF-α) and reducing the production of neurotrophins, such as brain-derived neurotrophic factor (BDNF) (Harry et al., 2006; Gibney et al., 2013). Neurotrophins, especially BDNF, are critical mediators of neuronal survival, and their altered levels have been linked to several neurodevelopmental disorders (Manti et al., 2023). Cytokines and active microglia also have a role in astrogliosis: proliferating astrocytes produce pro-inflammatory factors that may impact synaptogenesis, particularly inducing a preferential loss of inhibitory γ-aminobutyric acid (GABA)ergic synapses. This excitatory/inhibitory imbalance has been previously reported in neuropsychiatric disorders, such as ASD and epilepsy (Henstridge et al., 2019; Woods et al., 2023).

Additionally, MIA has been shown to alter the transplacental transportation of essential amino acids (i.e., leucine) (Kowash et al., 2022). In the human placenta, leucine and tryptophan are transferred from the mother to the fetus by the same transporter (Cleal and Lewis, 2008). Essential amino acids are precursors of neurotransmitters that are fundamental in neurodevelopment. Particularly, through the placental tryptophan hydroxylase activity, the placenta represents an important source of serotonin during the first trimester of pregnancy, influencing cortical neurogenesis, migration and initial axon targeting (Bonnin et al., 2011).

A recent study conducted by Perez-Palomar and colleagues in a rodent model proved that MIA disrupts dopaminergic and noradrenergic pathways in the prefrontal cortex, affecting cognitive behavior in offspring. These circuitries are notably impaired in schizophrenia (Perez-Palomar et al., 2023).

Several animal models and epidemiological studies have investigated the association between MIA and neuropsychiatric disorders, including schizophrenia, bipolar disorder, ADHD, cerebral palsy, developmental delay, cognitive dysfunction, anxiety/depression, and ASD (Garbett et al., 2012; Gibney et al., 2013; Canetta et al., 2014; Knuesel et al., 2014; Jiang et al., 2016; Han et al., 2021). It has been hypothesized that inflammatory responses to insults, such as respiratory infections and autoimmunity, might cause alterations in neural activity and brain connectivity (Kwon et al., 2022). Indeed, high amniotic interleukin concentration has been associated with preterm delivery, which is a risk factor for adverse neurodevelopmental outcomes (Hillier et al., 1995; Saigal and Doyle, 2008; Dicanio et al., 2022). Recent research proved that MIA can affect the neuropsychiatric outcomes of the offspring, even in asymptomatic mothers with increased intra-amniotic inflammatory cytokines levels (Gervasi et al., 2022). Noteworthy, neuropsychiatric disorders associated with MIA have heterogeneous clinical phenotypes. Therefore, some authors suggested that neurodevelopment is influenced by multiple risk factors and that MIA, by regulating gene expression and disrupting the neuronal differentiation and arborization, represents only one of the numerous “hits” that intervene in the onset of these conditions (Garbett et al., 2012; Woods et al., 2023).

## 5 The impact of prenatal influenza infection on neuropsychiatric outcomes in offspring

Influenza virus is an enveloped single-stranded RNA virus belonging to the *Orthomyxoviridae* family, which is classified into three genera (A, B, and C types) based on its antigenic profile. A further subdivision in serotypes takes into account the surface glycoproteins, such as hemagglutinin (HA) and neuraminidase (NA) (Beigi, 2014). All Influenza viruses have been implicated in human disease, and especially Influenza A and B, thanks to the antigenic drift (variation) of HA and NA, are responsible for annual epidemics with seasonal peaks in fall and winter (Manti et al., 2022). Conversely, the antigenic shift occurs when two or more different Influenza strains combine to produce a novel antigenic structure. This process happens approximately every 20–30 years and has been reported in Influenza A virus, the only type associated with Influenza pandemics until now (Beigi, 2014; Zimmer et al., 2021).

**Figure 3:**
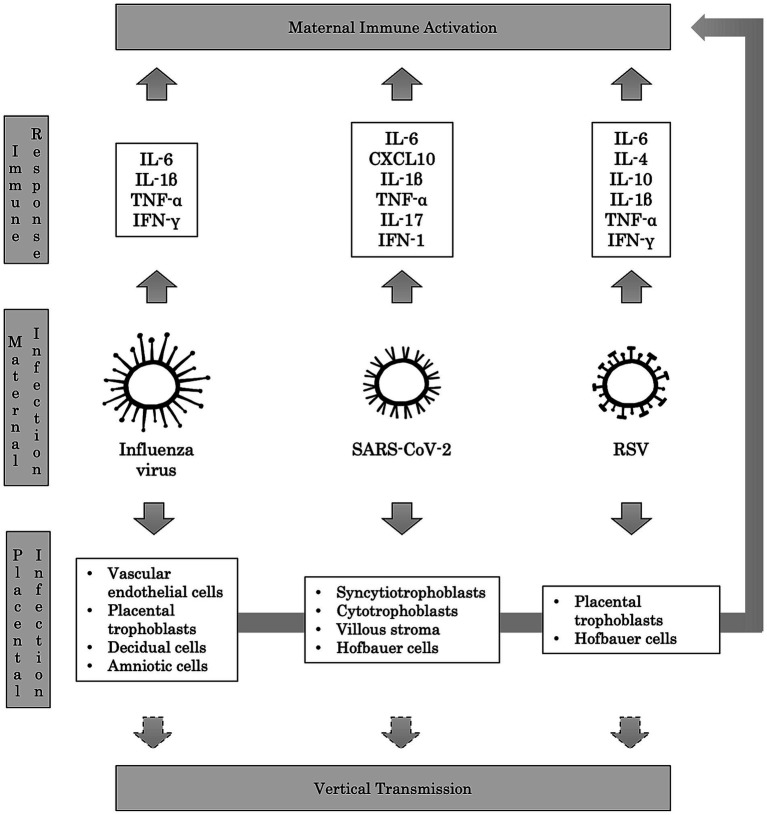
Immunopathological mechanisms of maternal respiratory infections. Maternal respiratory infections caused by Influenza virus, SARS-CoV-2, and RSV trigger the host’s immune response, leading to the production of several pro-inflammatory cytokines. This Maternal Immune Activation (MIA) is deemed responsible for adverse neuropsychiatric outcomes in offspring. Passing the viruses in the maternal blood may result in placental infection, potentially leading to vertical transmission to the fetus. In turn, the invasion of placental cells can provoke further production of pro-inflammatory cytokines, contributing to MIA.

Influenza virus can affect the feto-placental unit both directly and indirectly. The virus can induce the apoptosis of chorion by a direct cytopathic effect, and it can reach the placenta and the amniotic fluid through the maternal bloodstream, causing chorioamnionitis with degeneration of vascular endothelial cells, placental trophoblasts, decidual and amniotic cells (Manti et al., 2022). Moreover, the maternal immune response to the viral infection has been implicated in the association between Influenza and neuropsychiatric disorders. Indeed, it may lead to vascular inflammation and the production of inflammatory mediators such as IL-6, IL-1β, TNF-α, and IFN-γ, with detrimental consequences on brain development (Kwon et al., 2022) (Figure 3).

The association between the Influenza virus and neuropsychiatric disorders has long been studied in the literature, since Menninger - 1926 - reported an increase in influenza psychoses that looked like schizophrenia after the 1918 Influenza epidemic (Torrey and Peterson, 1976). Afterwards, epidemiologic studies confirmed a 3-fold increased risk of developing schizophrenia following Influenza exposure during the first half of gestation; the risk increased to 7-fold whether the infection occurred during the first trimester of pregnancy (Brown and Derkits, 2010). Moreover, research on the seasonality of birth in patients affected by schizophrenia and autism suggested a possible correlation with Influenza infection during the prenatal age (Zerbo et al., 2011; Fatemi et al., 2012; Zimmer et al., 2021).

Numerous animal models have been developed to explore better the correlation between the Influenza virus and the onset of neuropsychiatric disorders. Shi and colleagues administered intranasal Influenza virus on embryonic day 9.5 to pregnant mice. The authors demonstrated that the offspring showed behavioral abnormalities reminiscent of autism and schizophrenia (i.e., decreased sensitivity to auditory stimulation, increased anxiety, social deficits, and repetitive behaviors), some of which improved after antipsychotic drug treatment. Authors also suggested that Influenza infection contributed to the thinning of the neocortex and hippocampus, pyramidal cells atrophy, and reduction of reelin, a molecule responsible for proper laminar organization of the brain (Shi et al., 2003). In subsequent work, Fatemi and colleagues confirmed this evidence and hypothesized that prenatal Influenza infection might disrupt structure and gene expression in the placenta, as well as in the hippocampus and the prefrontal cortex of the offspring (Fatemi et al., 2012). Moreover, in a rodent model of Influenza infection, Garbett and colleagues highlighted alterations in gene expression involved in neurogenesis and neuroprotection in the acute phase of infection (Garbett et al., 2012). In the study conducted by Short and colleagues on Rhesus monkey, mothers’ infection during pregnancy also reduced early social interaction between the offspring and the mother, and alterations in neuroimaging were comparable to those shown in schizophrenic and autistic patients. Specifically, a significant reduction in brain volume has been described in the grey (prefrontal, frontal, cingulate, insula, parietal, and temporal-auditory regions) and in the white matter (parietal lobe) (Short et al., 2010).

The association between the Influenza virus and increased risk of neuropsychiatric conditions different from schizophrenia has also been investigated. Particularly, Parboosing and colleagues showed a 6-fold increased risk of developing bipolar disorder after exposure to maternal Influenza during pregnancy in a population-based cohort study (Parboosing et al., 2013). In a successive study on the same cohort, the authors assessed the difference between patients with bipolar disorder and patients with bipolar disorder and psychotic symptoms. They found a 5-fold increased risk of bipolar disorder with, but not without, psychotic features. Therefore, they proposed that maternal Influenza exposure was a risk factor for psychosis, rather than for a specific psychotic disorder diagnosis, influencing the same neuronal circuitries of schizophrenia (Canetta et al., 2014). These data were confirmed by a recent systematic review conducted by Fung and colleagues, which confirmed an increased risk for schizophrenia and other primary psychotic disorders in children exposed to maternal Influenza infection during pregnancy, especially during the first and second trimesters. Moreover, the authors reported an increased risk of developing ASD in offspring exposed to maternal Influenza infection throughout the whole gestation (Fung et al., 2022).

However, the Influenza virus has already been proven to impact neurodevelopment in the early post-partum period. In a population-based cohort study, Borren and colleagues reported slightly poorer psychomotor development scores in 6-month-old children whose mothers had Influenza symptoms during pregnancy. The difference became significant when considering the fine-motor alterations in children with early exposure to the Influenza virus (within the 8th week of gestation) and the communication subdomain in infants with later exposure (9–40th weeks of gestation). This evidence confirmed the impact of maternal Influenza infection on the cognitive and social abilities of the offspring (Borren et al., 2018).

## 6 The impact of prenatal SARS-CoV-2 infection on neuropsychiatric outcomes in offspring

SARS-CoV-2 belongs to the *Coronaviridae* family, comprising six genera, three of which include viruses potentially infecting humans (Zimmer et al., 2021). It is an enveloped RNA virus primarily transmitted via respiratory droplets. After infecting the nasal mucosa, it can be transmitted to lower airway cells that co-express angiotensin-converting enzyme 2 (ACE2) and transmembrane serine protease 2 (TMPRSS2). Moreover, SARS-CoV-2 infection induces an abnormal systemic inflammatory response (cytokine storm) that may lead to acute respiratory distress syndrome by producing IL-6, C-X-C motif chemokine 10 (CXCL10), and INF-1 (Tay et al., 2020).

Placental expression of ACE2 mRNA is gestational-age dependent, with the highest levels in early pregnancy, and viral proteins and/or viral RNA have been detected in syncytiotrophoblasts, cytotrophoblasts, villous stroma, and possibly Hofbauer cells (Li et al., 2020; Fahmi et al., 2021; Lye et al., 2021). Therefore, vertical transmission has been hypothesized, and it has been estimated as 3.67% in newborns of SARS-CoV-2-positive mothers (Dube and Kar, 2020; Fenizia et al., 2020). Several studies showed that neurons, microglia, and NPCs might express ACE2 and TMPRSS2, allowing the infection of the developing brain (Cui et al., 2019; Baig et al., 2020; Yang et al., 2020; Tiwari et al., 2021). Remarkably, in a recent study, expression of ACE2 mRNA was detected in circulating granulocytes, monocytes, and lymphocytes from pregnant women with chorioamnionitis, proving an increased risk of SARS-CoV-2 vertical transmission (Lye et al., 2021). However, it has been proposed that the damage to the fetal brain due to SARS-CoV-2 infection is mainly caused by vascular injury, leading to placental ischemia and inflammation (Sharps et al., 2020; Lye et al., 2021; Liu et al., 2022). In fact, MIA response to SARS-CoV-2 infection is considered the main mediator of the neurodevelopmental disruption in the fetus (Woods et al., 2023). Particularly, increased levels of IL-1β, IL-6, IL-17, and TNF-α have been detected in the peripheral blood of pregnant women compared to controls (Fenizia et al., 2020; Figure 3).

Given the recent onset of the COVID-19 pandemic, no studies address the long-term consequences of maternal infection on the offspring’s neuropsychiatric outcome. Nevertheless, abundant research has been conducted to explore the effects of SARS-CoV-2 on neurodevelopment (San Martín-González et al., 2023). Particularly, Aldrete-Cortez and colleagues examined the general movements of 28 3-to5-months-old children exposed to SARS-CoV-2 infection during the third month of gestation compared to the same number of controls. The exposed children showed significantly poorer motor performances and higher rates of absent or abnormal fidgety movements, proving an increased risk of adverse neurodevelopmental outcomes (Aldrete-Cortez et al., 2022). General movements assessment, particularly fidgety movements, has been extensively used in the literature to predict cerebral palsy since it showed a sensitivity of 98% and a specificity of 91%. However, atypical fidgety movements have also been associated with cognitive impairment, ADHD, and ASD (Hadders-Algra, 2021).

Early fine motor abnormalities have been described by Liu and colleagues at the age of 1–2 months in the offspring of a mother to SARS-CoV-2 during pregnancy. However, these abnormalities were not reported at 12–13 months, after a parent-guided postnatal training. This data shows that a precocious intervention could correct neurodevelopmental delay (Liu et al., 2022). In the study conducted by Cheng and colleagues, the fine motor abnormalities were significantly higher in 9-month-old children exposed to prenatal maternal infection compared to non-exposed children. The authors also found lower scores in communication, gross movement, problem-solving, and personal-social domains, yet not statistically significant (Cheng et al., 2021). Similarly, Ayed and colleagues conducted a prospective cohort study on 298 children born to mothers with SARS-CoV-2 infection during pregnancy and reported a 10% of developmental delay at the age of 10–12 months, with a prevalent deficit in fine motor abilities. Noteworthy, the developmental delay was more common when infection occurred during the first and second trimesters of pregnancy (Ayed et al., 2022). Additionally, Mulkey and colleagues reported an increased risk of developmental delay related to maternal symptomatology. Children born to mothers with symptomatic SARS-CoV-2 infection were found to be at a higher risk of having lower developmental scores, especially in fine motor and personal-social domains. These infants also showed mild truncal hypotonia, which can be associated with lower motor skills (Mulkey et al., 2022).

On the contrary, Shuffrey and colleagues did not find significant differences among their cohort of infants exposed to maternal SARS-CoV-2 infection during pregnancy and children born before the pandemic. However, the authors observed that infants born during the pandemic obtained significantly lower scores on the gross motor, fine motor, and personal-social subdomains, irrespective of maternal infection. Therefore, they suggested that COVID-19-related stress should be considered a potential mechanism underlying the neurodevelopmental abnormalities experienced by these children (Shuffrey et al., 2022). Similarly, Wu and colleagues reported significantly lower scores in fine motor, problem-solving and personal-social subdomains in a cohort 3-month-old infants with prenatal exposure to SARS-CoV-2; however, the social–emotional and overall developmental delay did not correlate to the maternal infection. Interestingly, the authors found a negative correlation between lower gross motor scores and the length of mother-infant separation after birth (Wu et al., 2021). It is worthy of note that the last two studies had few cases of infection during the first trimester, which is considered by some authors the most critical period for fetal infection (Li et al., 2020; Lye et al., 2021; Wu et al., 2021; Shuffrey et al., 2022). Nevertheless, children exposed to third-trimester maternal infection have also reported an increased risk for neurodevelopmental disorders. Edlow and colleagues, in a retrospective cohort study performed on 222 newborns of mothers infected with SARS-CoV-2, observed higher rates of neurodevelopmental disorders compared to children delivered in the same period. The majority of these diagnoses reflected developmental disorders of motor function or speech and language (Edlow et al., 2022).

Besides the neurodevelopmental impairment, sparse cases of children with neonatal ischemic brain lesions correlated with SARS-CoV-2 maternal infection have been reported in the literature. The patients mainly showed thalamic infarction, though alterations of the white matter have also been described (Vivanti et al., 2020; Beslow et al., 2021; Engert et al., 2021; Brogna et al., 2022; Campi et al., 2022). Placental inflammation, vascular endothelial damage, and consequent activation of the clotting cascade that induces thrombotic events have been proposed as pathogenic mechanisms involved in neurodevelopmental impairment (Sharps et al., 2020; Lye et al., 2021; Brogna et al., 2022; Liu et al., 2022).

## 7 The impact of prenatal RSV infection on neuropsychiatric outcomes in offspring

Respiratory syncytial virus (RSV) is an enveloped single-stranded RNA virus of the *Paramyxoviridae* family (Keyserling, 1997). The surface glycoproteins G (Ga and Gb) identify two subtypes, namely A and B; together with the surface glycoprotein F (fusion). These proteins facilitate the interaction with the host airway epithelial cells, merge the viral envelope to the membranes of multiple adjacent cells, and form the “syncytia.” Thus, they appear crucial for the virulence of RSV and the principal antigens presented to the host immune system (Piedimonte, 2015; Andeweg et al., 2021).

RSV causes annual epidemics lasting 2–5 months and it is the primary cause of hospitalization in the pediatric population within 6 months of age; by the age of 2 years, most infants have been infected at least once by RSV and reinfected in 40% of the cases (Keyserling, 1997; Kazakova et al., 2019; Andeweg et al., 2021; Manti et al., 2023). However, it is among the most common pathogens causing acute respiratory tract infections, even in adulthood. Its disease burden has been estimated to be similar to that of Influenza A in high-risk populations (Falsey et al., 2005; Nam and Ison, 2019). Pregnant women are more susceptible to respiratory infection, and RSV is responsible for up to 10–11% of this population’s acute respiratory tract infections (Beigi, 2014; Wheeler et al., 2015; Hause et al., 2019).

Several animal models have proven vertical transmission of RSV (Piedimonte et al., 2013; Piedimonte and Perez, 2014; Brown et al., 2017; Manti et al., 2018; Piedimonte and Harford, 2020). Moreover, RSV has been identified at birth in an infant presenting with respiratory distress symptoms, born to a woman with an elevated titer of antibodies against RSV (Manti et al., 2017). Accordingly, in a clinical study conducted at birth on 22 newborns from mothers who contracted RSV during the third trimester of pregnancy, anti-RSV IgA, IgM, and IgG titers were found in the cord blood samples (Manti et al., 2020). All these data together enforce the assumption that RSV may reach the fetus via the placenta and subsequently affect brain development (Andrade et al., 2021; Figure 3).

In addition to respiratory symptoms, RSV showed a neurotropism as assessed by several studies showing that RSV infection is associated with neurological manifestations, such as seizures, status epilepticus, encephalitis, neonatal encephalopathy with SIADH, hemiconvulsion–hemiplegia syndrome, persistent lethargy, feeding or swallowing difficulties, abnormalities of tone, and strabismus (Hirayama et al., 1999; Ng et al., 2001; Kho et al., 2004; Otake et al., 2007; Millichap and Wainwright, 2009; Picone et al., 2019). Additionally, RSV RNA was detected in the cerebrospinal fluid of patients presenting with RSV infection and seizures (Zlateva and Van Ranst, 2004; Kawashima et al., 2009). Moreover, CSF analysis of patients with RSV-associated neurological abnormalities showed increased levels of inflammatory cytokines, particularly IL-6 (Otake et al., 2007; Kawashima et al., 2009).

Following these reports, emerging research focused on the mechanisms through which RSV infection might disrupt brain development. In an *in vivo* model, Espinoza and colleagues proved that RSV infection can reach the CNS via the hematogenous pathway associated with leukocytes. The authors suggested RSV may cause behavioral and cognitive impairment through reduced synaptic plasticity in the hippocampal regions (Espinoza et al., 2013). Similarly, Bohmwald and colleagues reported a significant impairment of exploratory behavior in RSV-infected mice compared to controls. They also found increased levels of inflammatory cytokines (i.e., IL-4 and IL-10) and glial fibrillary acidic protein (GFAP) mRNA levels in the infected mice. Therefore, the authors suggested these alterations may contribute to cognitive impairment, as observed in other preclinical studies regarding Influenza virus and SARS-CoV-2 (Bohmwald et al., 2021).

Although no study has been conducted on the relationship between RSV and MIA, alterations in proinflammatory cytokine levels and their effects on brain development have been investigated in other respiratory viruses (Andrade et al., 2021). Nevertheless, RSV showed a differential tropism for distinct human placental cells, particularly the Hofbauer cells, which in turn produced increased levels of proinflammatory cytokines (i.e., IL-6, TNF-α, and IFN-γ; Bokun et al., 2019). All this evidence supports the potential role of the maternal immune response against RSV as an additional mechanism in disrupting brain development.

## 8 The impact of other prenatal respiratory infection on neuropsychiatric outcomes in offspring

Other respiratory viral infections during pregnancy have been related to adverse neuropsychiatric outcomes in the offspring.

SARS-CoV-1 and Middle East respiratory syndrome (MERS) showed a weak neurotropism, and some reports of related neuropsychiatric sequelae had been described in infected adults, though infection complications in the offspring of affected mothers have not been investigated (Cheng et al., 2004; Arabi et al., 2015; Zimmer et al., 2021).

On the contrary, rubella’s effects on neurodevelopment have been widely investigated. Rubivirus is an enveloped single-stranded RNA virus belonging to the Matonaviridae family, and it is known to infect the fetal brain directly, causing neuronal death, gliosis, and a disruption of mitosis (Brown et al., 2000; Ejaz et al., 2022). Congenital rubella syndrome includes systemic symptoms, such as cataracts or congenital glaucoma, congenital heart disease, hearing impairment, and pigmentary retinopathy (Gordon-Lipkin et al., 2021). However, maternal infection may also cause neuropsychiatric conditions in the offspring. Brown and colleagues observed an increased rate of nonaffective psychosis in patients exposed to rubella during pregnancy (Brown et al., 2000). Following the 1964 pandemic, Chess found an increased prevalence of ASD in a retrospective observational study including 243 children who had been affected by rubella *in utero* (Chess, 1971). Moreover, a 10-to-15-fold increased risk of developing schizophrenia spectrum disorders and a decline in intelligence quotient between childhood and adolescence have been reported (Zimmer et al., 2021).

Finally, some reports in the literature examined the adverse outcome of respiratory viral infections without specifying the etiologic agents. Freedman and colleagues evaluated children born to mothers affected by upper respiratory infections during pregnancy. The authors reported a worse ability to maintain attention and bond to their parents when maternal choline levels were lower than 7.5 μM, suggesting a protective role of choline in fetal behavioral development, even if the viral infection occurred in early gestation (Freedman et al., 2020). Bear and colleagues performed a retrospective cohort study to analyze the association between gestational infections and cerebral palsy. They found a 2-fold increased risk of developing cerebral palsy in children whose mothers suffered from respiratory illness during pregnancy; the risk raised to almost 3-fold if the infection was diagnosed in the third trimester (Bear and Wu, 2016).

## 9 Conclusion

The physiological modifications occurring in pregnant women allow the survival of the fetus but increase susceptibility to infections and adverse offspring outcomes (Bear and Wu, 2016; Zimmer et al., 2021; Manti et al., 2022). The gestational age is a period featuring significant growth and changes in the CNS, making the brain vulnerable to intrauterine environmental insults throughout a protracted developmental window and with potentially long-lasting effects (Davis et al., 2011). A possible role of infection in the aetiology of psychotic diseases has been hypothesized for over a century (Torrey and Peterson, 1976; Khandaker et al., 2013). To date, a strong association between neuropsychiatric disorders and prenatal viral infections, especially respiratory viruses, has been outlined (Zimmer et al., 2021; San Martín-González et al., 2023).

Respiratory viruses can interfere with CNS development both directly, through vertical transmission, and indirectly, by inducing placental hypoxia and affecting its physiologic functioning (Lye et al., 2021; Liu et al., 2022; Manti et al., 2022). Additionally, numerous studies investigated the possible role of MIA and cytokines effects on the developing brain, resulting in lifelong neurodevelopmental disorders. The disproportionate maternal immune response can interfere with the placental capacity to provide nutrients and crucial precursors for normal fetal development. Furthermore, cytokines contribute to placental regulation, and their excessive production has been implicated in the dysfunction of developmental processes and derangement of physiological fetal homeostasis (Knuesel et al., 2014; Woods et al., 2023). Respiratory viral infections have been frequently reported in pregnant women, and the recent pandemics raised some concerns regarding the neurodevelopmental outcomes of the children born to these mothers (San Martín-González et al., 2023; Sturrock et al., 2023). However, they offered a remarkable opportunity to understand better the effects of prenatal infections on the offspring’s neurodevelopmental outcomes.

Particularly, the association between the Influenza virus and neuropsychiatric disorders has been extensively studied throughout the last century, proving an increased risk for conditions involving cognitive and social abilities in children and adults (i.e., schizophrenia, autism, and bipolar disorder with psychotic symptoms). Data from recent systematic reviews indicate that exposure to Influenza may be more harmful during the early stages of gestation (Torrey and Peterson, 1976; Brown and Derkits, 2010; Zerbo et al., 2011; Fatemi et al., 2012; Khandaker et al., 2013; Canetta et al., 2014; Zimmer et al., 2021; Fung et al., 2022).

On the contrary, given the recent onset of the COVID-19 pandemic, the literature addressing neuropsychiatric consequences of the SARS-CoV-2 infection during pregnancy was mainly focused on early infancy. Overall, an impairment of the fine motor abilities, associated or not with global developmental delay, is the most reported outcome in children born to mothers affected by SARS-CoV-2 during gestation (Cheng et al., 2021; Aldrete-Cortez et al., 2022; Ayed et al., 2022; Liu et al., 2022; Mulkey et al., 2022). Moreover, though not statistically significant, lower scores in the personal-social domains have been reported in several researches (Cheng et al., 2021; Wu et al., 2021; Mulkey et al., 2022; Shuffrey et al., 2022).

Despite the scarcity of literature regarding the neuropsychiatric disorders that could affect offspring born to mothers with prenatal RSV infection, consistent data reported neurological sequelae in children with RSV infection in the perinatal epoch or during the early months of life (Ng et al., 2001; Kho et al., 2004; Millichap and Wainwright, 2009; Picone et al., 2019). Emerging findings support the capacity of RSV to cross the placental-fetal interface, inducing respiratory conditions overlapping the symptoms of a postnatal RSV infection (Manti et al., 2017, 2020). Therefore, it is reasonable to hypothesize that neurological complications could also manifest after RSV infection during pregnancy, and the possible consequences should also be considered.

Early identification of brain developmental trajectories in children linked to maternal respiratory infections is crucial to conceiving preventive strategies for clinical outcomes. In light of the above data, vaccinations, antiviral therapy, and immune modulators are urgently required to minimize maternal infection, fetal transmission, and an “inappropriate” excessive immune response. It is worth considering that potent adjuvants could affect the cytokine balance at the fetal–maternal interface (Gerdts et al., 2016); therefore, inactivated vaccines against the most common viral agents before and during the pregnancy have been considered safe and strongly recommended (Knuesel et al., 2014). Very recently, the US Food and Drug Administration approved the administration of an RSV vaccine to pregnant people as a single injected dose during the third trimester (Harris, 2023). Especially during the early gestation stages, when the offspring CNS is more vulnerable to infectious triggers, vaccinations and monoclonal antibodies could provide adequate protection for the mother and the fetus, leading to better short- and long-term outcomes (Manti et al., 2022).

In parallel, recognizing altered developmental trajectories allows a prompt intervention that can improve the neurodevelopmental outcome of children (Liu et al., 2022). Accordingly, neuropsychiatric follow-up is advisable in newborns of mothers affected by respiratory infections during pregnancy. Finally, more research is necessary to assess the long-term consequences of SARS-CoV-2 infection during pregnancy on the offspring’s neuropsychiatric outcome and to detect adverse neurological manifestations possibly related to RSV maternal infection.

## Author contributions

SM: Data curation, Methodology, Resources, Visualization, Writing – review & editing. GS: Data curation, Methodology, Software, Visualization, Writing – original draft, Writing – review & editing. AN: Data curation, Software, Validation, Visualization, Writing – review & editing. GR: Validation, Visualization, Writing – review & editing. GP: Conceptualization, Funding acquisition, Resources, Supervision, Validation, Visualization, Writing – review & editing.
